# Physical restraint of children and adolescents in mental health inpatient services: A systematic review and narrative synthesis

**DOI:** 10.1177/1367493520937152

**Published:** 2020-07-07

**Authors:** Simon Nielson, Lucy Bray, Bernie Carter, Joann Kiernan

**Affiliations:** Faculty of Health and Social Care, 6249 Edge Hill University, UK

**Keywords:** Aggression, child and adolescent mental health services, environment, physical restraint, restrictive interventions

## Abstract

Physical restraint is regularly used in children and adolescent mental health care, often as a reactive behaviour management strategy. Physical restraint has been associated with physical injury, but psychological consequences are poorly understood. The aim of this systematic review was to examine physical restraint of children and adolescents in inpatient mental healthcare services. Healthcare databases were searched to identify English language publications discussing anyone aged ≤18 years who had experienced physical restraint as a mental health inpatient. No date restrictions were applied. Sixteen quantitative studies are included within this review. Most studies are retrospective in nature. Publications were appraised using the Critical Appraisal Skills Programme quality assessment tool. Common characteristics associated with children and adolescents who experience physical restraint include age, gender, diagnosis, and history. Most studies associate physical restraint with the management of aggression. Findings suggest that it may be a combination of patient (intrinsic) and environmental (extrinsic) factors which ultimately lead to children and adolescents experiencing restraint. This review confirms that little is known about children and adolescents’ first-hand experiences of physical restraint. Future research should address children and adolescents’ perceptions and first-hand experiences of physical restraint.

## Introduction

Restrictive interventions, which include restraint, have a long history within mental healthcare services. Physical restraint in mental health care means ‘…the use of physical contact which is intended to prevent, restrict, or subdue the natural movement of any part of the patient’s body’ (Mental Health Units (Use of Force) Act, 2018). Despite the potential risk of physical injury as a result of being physically restrained (Department of Health, 2014), its implementation in mental healthcare practice is sometimes deemed necessary to maintain safety (Wilson et al., 2015) and protect children and adolescents and/or people around them from harm (National Institute for Health and Care Excellence, 2017). However, there are ethical, moral, and legal considerations associated with its implementation in all fields of health care (Hollins, 2017), including disproportionate use (Georgieva et al., 2012), unnecessary exposure to injury (Hollins and Stubbs, 2011), and death (Barnett et al., 2012; Scheuermann et al., 2016; United Nations Convention on the Rights of the Child, 1989). The focus on physical risks can result in psychological harm being overlooked (Bray et al., 2014; Ridley and Leitch, 2019).

In the United Kingdom, ‘…physical interventions should only ever be used as a last resort…’ (Department of Health, 2014: 9) and broad guidelines, targeted at health, social care, and educational providers, as well as local authorities and clinical commissioning groups, aim to eliminate the inappropriate use of physical restraint with children and adolescents who are ‘…still developing both physically and emotionally and for whom trauma…could be very damaging…’ (Department of Health, 2017: 5). However, historically, not all healthcare guidelines have provided evidence-based frameworks of how to implement standards (Wilson et al., 2015).

Understanding the consequences of physical restraint on a child’s mental and physical well-being is important (Ridley and Leitch, 2019). Further, research indicates that adults (patients and staff) can also experience distress (Tolli et al., 2017), anger, fear (Merineau-Cote and Morin, 2013), anxiety (Laugharne et al., 2012), and traumatic psychological damage (Mohr et al., 2003) resulting from restraint implementation. There are also reports that the use of restraint within mental health services can damage therapeutic rapport (Steinert et al., 2010) and contribute to increased staff turnover (Department of Health, 2017). An important resource for service providers and policy-makers, both in the United Kingdom and internationally, would be access to a contemporary systematic literature review on the use of physical restraint with children and adolescents within mental health settings. Currently, this does not exist, and this review aims to address this deficit.

## Aim of the review

The aim of this review was to systematically locate, appraise, analyse, and synthesise literature pertaining to the physical restraint of children and adolescents in inpatient mental healthcare services. The objectives are to

1. explore which children and adolescents are being physically restrained in inpatient mental health services,
2. critically examine the reasons why children and adolescents are being physically restrained in inpatient mental health services, and
3. critically examine the consequences for children and adolescents of reported physical restraint use in inpatient mental health services.

## Method

### Selection of a narrative approach

Due to the heterogeneity of the combination of sample populations and settings (Popay et al., 2006) as well as the poorly understood nature of physical restraint of children and adolescents in mental health care, a narrative synthesis approach was chosen (Campbell et al., 2018), informed by Popay et al. (2006), to ‘tell the story’ of findings from the included quantitative studies (page 5).

### Search criteria

The review was conducted in accordance with the Preferred Reporting Items for Systematic Reviews and Meta-Analyses statement. A comprehensive search identified relevant peer-reviewed research publications. Databases included ProQuest, CINAHL, the Cochrane Library, PubMed, MEDLINE, and PsycINFO. A modified version of the PICO tool focused the search using combinations of words associated with the population (children and adolescents), the concept (physical restraint), and the context (inpatient mental health care) (PCC) (Table 1). The search was completed in October 2018.

**Table 1.** Key search terms.

Population (P)	Adolescen*, Child*, P?ediatri*, Teen*, Young pe*, and Youth*
Concept (C)	Behavio?r control, Behavio* management, Clinical* hold*, Hold*, Immobili*, Physical* interven*, Physical* restrain*, Restrain*, Restrict* practice*, Restrictive intervention*, and Therapeutic* hold*
Context (C)	CAMH*, Child* and adolescent* mental health, Inpatient CAMH*, P?cediatric assessment unit*, and Tier 4 CAMH*

*Note:* CAMH: child and adolescent mental health.

### Inclusion and exclusion criteria

The eligibility criteria included empirical studies, availability in English, and reporting on physical restraint use with children and adolescents admitted to mental health services. Studies were excluded if physical restraint was not independently reported from seclusion (a restrictive intervention which falls beyond the remit of the current study). Due to the paucity of the literature, no date restrictions were applied.

### Screening

**Figure 1.**
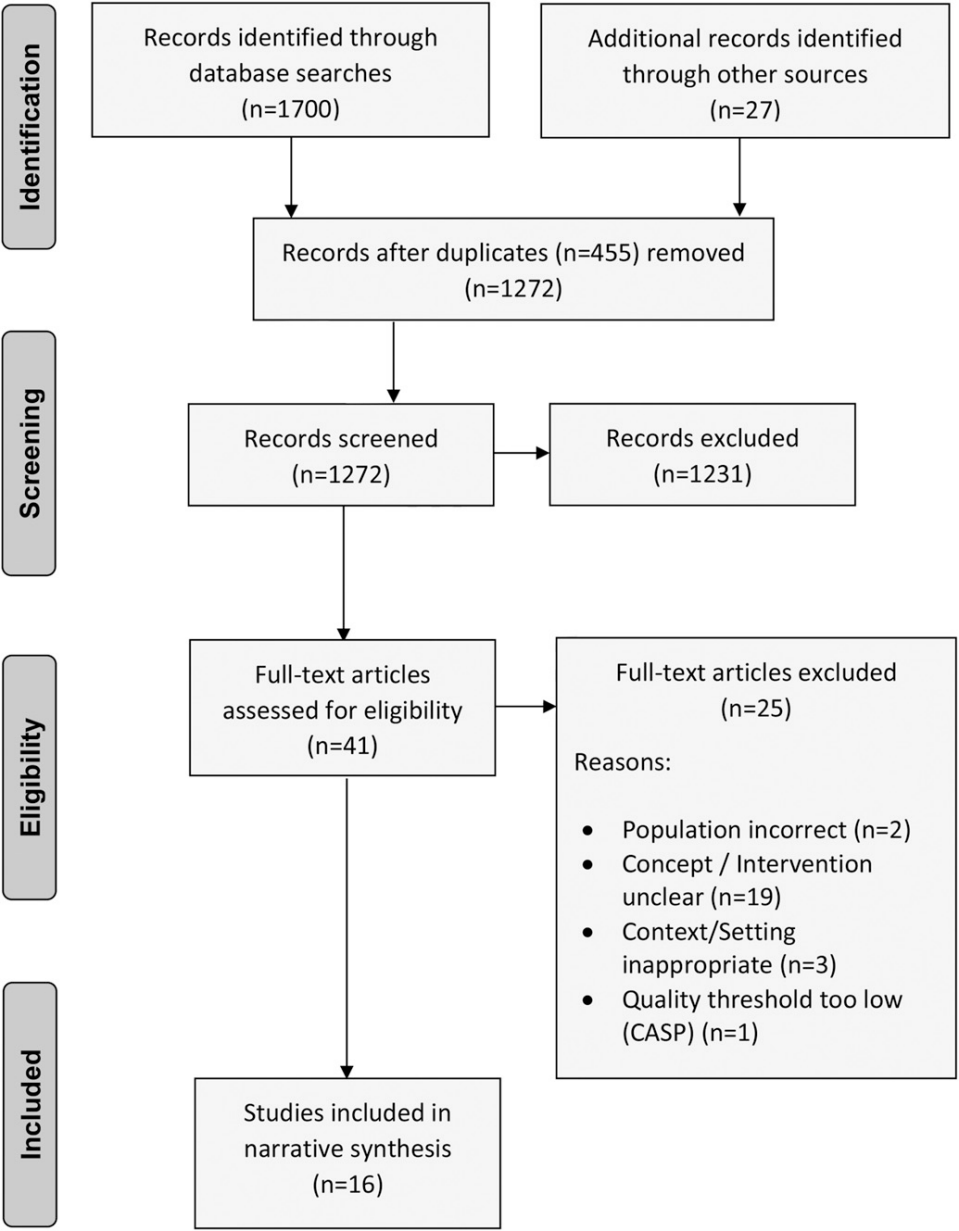
Preferred reporting items for systematic reviews and meta-analyses flowchart.

The initial searches retrieved 1727 publications. Of those, 455 duplicates were removed. Titles, keywords, and abstracts of all remaining publications (*n* = 1272) were screened for relevance, and 1231 publications were excluded (Figure 1). Reference lists and lead authors of the remaining publications (*n* = 41) were examined to identify relevant additional studies. Full-text screening resulted in a further 24 publications being excluded. Data were then extracted from the remaining publications (*n* = 17); in Table 2, these studies are presented in order of publication.

**Table 2.** Included studies (ordered by year of publication).

Author(s), year, country	Aim	Design/method	Population	Outcome measure	Finding	Limitation	Reviewer comment
Sourander et al. (2002), Finland	To analyse the use of holding, restraints, seclusion, and time-out in child and adolescent psychiatric inpatient treatment	Retrospective exploratory study	Children and adolescent inpatients (2–18 years of age) (*n* = 504)	Use of time-out	In univariate analysis, younger age (OR 6.2, 95% CI 3.9–9.8), male gender (OR 3.0, 95% CI 1.9–4.9), aggressive acts (OR 11.2, 95% CI 7.0–17.8), conduct/oppositional disorder (OR 1.7, 95% CI 1.1–2.6), attention deficit hyperactivity disorder (OR 3.8, 95% CI 1.7–8.7), attachment disorder (OR 3.5, 95% CI 1.5–8.1), autism spectrum disorder (OR 4.5, 95% CI 2.0–9.9), and longer length of stay (OR 2.8, 95% CI 1.8–4.3) were significantly associated with physical holding	No information about the efficacy of techniques was collected	No discussion about transferability of findings although the authors recognise that that it would be important to compare the different practices and legal issues in different EU countries and work towards the formulation of common restraint guidelines
				Use of therapeutic holding			
				CGAS			
				Diagnostic grouping			
				School social behaviour scale			
				Short adaptive behaviour scale			
Donovan et al. (2003), USA	To examine demographic characteristics associated with the use of seclusion and restraint among psychiatrically hospitalised youths	Retrospective naturalistic study	Children and adolescent inpatients (5–18 years of age) (*n* = 442)	Anonymised demographic data	Children and adolescents who were admitted on an emergency basis and those belonging to ethnic minority groups were more likely to undergo seclusion or restraint	Although data show a clear downward trend in the use of restraint, the reasons could not be addressed by this observational study	This study examined data from a single public institution; as such, it may not be representative
				Anonymised clinical data		Short physical holds (less than 15 min), ‘time-outs’, ‘escorts’, and ‘basket holds’ were not included in the dataset	This study uses a comparatively weak retrospective methodological design
				Incident forms		There was a paucity of qualifiers regarding the nature and severity of injuries and the characteristics of the as-needed (pro re nata) medications used	
Delaney and fogg (2005), USA	To examine the characteristics of children and adolescents who were restrained during brief inpatient psychiatric treatment	Retrospective study	Children and adolescent inpatients (7–19 years of age) (*n* = 100)	Medical charts	Children and adolescents were significantly more likely to be restrained if they were male, had multiple admissions, had longer length of stay, had a psychotic disorder diagnosis, or had previous hospitalisations	This was a small, single-site study, which limits generalisations	This study uses a comparatively weak retrospective methodological design
				Incident forms	Restrained children and adolescents were more likely to have a history of voicing/attempting suicide	Lack of associated data made some study variables difficult to interpret	
				Chart audit forms		Without a severity of illness score, explanations were limited for the reason for multiple restraints	
				Aggression rating scale			
Leidy et al. (2006), USA	To explore the nature of restraints with female adolescents in residential treatment facilities	Retrospective exploratory study	Female children and adolescent inpatients (11–17 years of age) (*n* = 415)	Clinical case files	The length of the restraints ranged from 1 min to 98 min	There were other child and staffing variables that were not considered due to lack of resources and the capacity to extract relevant data	The aim and methodology of this study could be clearer
				Anonymised demographic database	77% of restraints were in the late afternoon/evening		There is no clarity over who was accessing non-anonymised data before the anonymised databases were created
				Restraint database	Restraints tended to cluster. When one occurred, another often shortly followed		
Crocker et al. (2010), Australia	To determine the predictors of aggression and restraint in children admitted to a child psychiatric inpatient unit	Retrospective exploratory study	Children and adolescent inpatients (5–12 years of age) (*n* = 41)	Incident reports	A high-risk assessment was associated with aggressive behaviour and subsequent restrain	This was a single-site, retrospective audit, with a small sample size and low statistical power	The authors could have strengthened their position by comparing characteristics of their non-violent study population (*n* = 29)
				Risk assessment rankings	Disruptive behaviour disorder predicted more serious aggression leading to restraint	There is an increased risk of error as an alpha value of 0.05 was used during analysis	The process of clinical data extraction (and error checking procedure) is not reported and has the potential to carry some level of subjectivity
						Staff may not have completed the incident forms uniformly	
						Data needed to be categorised, and the cut-off may have influenced findings	
dosReis et al. (2010), USA	To determine which time period following a residential admission would provide the best estimate of youth who are low, moderate, or high risk for future seclusion/restraint	Retrospective naturalistic study	Children and adolescent inpatients (12–18 years of age) (*n* = 298)	Medical records	544 (53%) interventions occurred after school*/*evening, 344 (33%) during school*/*daytime hours, 125 (12%) around bedtime*/*late evening, and 21 (2%) in the early morning	There is the likelihood of missing details with retrospective data	The sample size seems appropriate although there is no indication of the power of the sample
				Treatment plans	There was a steep increase in the seclusion*/*restraint procedures at month three	The inclusion of only patients in residence for 12+ months may have selected those who were more challenging	Data may not be generalisable
							It is unclear who was responsible for collecting the data
Stewart et al. (2010), Canada	To define which patients are at greatest risk for being administered intrusive interventions as a first step to reducing the need for this controversial practice	Retrospective analysis	Children and adolescent inpatients (infancy-18 years of age) (*n* = 153)	Child and adolescent functioning scale	54.63% of the sample received some form of intrusive intervention	The sample was disproportionately male	The authors provide a good statement on the need for a predictive model and support their analysis with a clear background review
				Brief child and family phone interview	Age at admission and sex reliably predicted administration of an intrusive intervention	Potential historic abuse/maltreatment was not considered	No indication of ethics approval, but the retrospective nature negates potential additional burden
				Intrusive intervention database	Self-harm demonstrated a trend towards significance (*p* = 0.057)	No controls were established for specific mental health diagnoses	
Azeem et al. (2011), USA	To determine the effectiveness of six core strategies based on trauma informed care in reducing the use of seclusion and restraints with hospitalised youth	Retrospective analysis	Children and adolescent inpatients (6–12 years of age) (*n* = 458)	Medical charts	Seventy-nine patients (17.2%) required 119 restraints, with an average number of episodes (3.5/patient) (range 1–28)	Data were not sufficient to express results as per thousand patient days	There are relatively small amount of baseline data
				Incident forms	The major diagnoses of the youth placed in seclusion and/or restraints were disruptive behaviour disorders (61%) and mood disorders (52%)	Effectiveness of strategies could have been biased by concurrent initiatives on the unit	Effectiveness could be better measured through a randomised control trial
						Longer baseline data prior to implementation were not available	The design is potentially too weak to draw any strong conclusion as to training-related effects
	

Tompsett et al. (2011), USA	To address the limitations of previous research by prospectively assessing theoretically defined predictors of aggression in an adolescent inpatient sample	Prospective translational action research	Children and adolescent inpatients (5–17 years of age) (*n* = 149)	Critical incident reports	Restraint resulting from patient-to-staff aggression was a most common predictor (*n* = 5145), with patient-to-patient (*n* = 520) and self-injurious behaviours (*n* = 530) less common	The outcome variable is only an approximation of aggression	The study does not attempt to compare across hospitals
				The risk analysis checklist for institutionalised youth		The risk analysis measure, conducted by therapists in treatment planning, is a significant limitation	There are unmeasured factors which may also be contributing to the likelihood of restraint
						Data validity is reliant upon verbalised reports which may contain inaccuracies	This study relies on limited outcome measures
Pogge et al. (2013), USA	To explore whether restraint and seclusion events are more common and more abbreviated in younger patients	Prospective comparison analysis	Patients on a child unit (12 years of age or younger) and patients on two adolescent units (13–17 years of age)	Global assessment of functioning scale	Younger patients experienced more restraint episodes	Data are from a single facility	No information is available concerning successful interventions that did not include restraint
				Derogatis psychiatric rating scale	None of the IQ index scores was significantly associated with restraint or seclusion (all t\1.15, all P[0.25)	The frequency of disruptive behaviour incidents was only recorded when a restraint or seclusion took place	
				Global perspective inventory			
				Children’s psychosocial rehabilitation scale			
				Wechsler intelligence scale for children			
Stewart et al. (2013), Canada	To identify some of the factors associated with the use of intrusive measures among children with mental health and developmental disabilities	Retrospective intervention study	Children and adolescent inpatients (6–18 years of age) (*n* = 338)	The child and adolescent functioning assessment scale	Children who were aged between 11 and 13 years were 33% less likely to have been physically restrained, and children aged 14 years and older were 68% less likely to have been physically restrained than children younger than 10 years	Participants were not randomly sampled	The interpretation and attempts to generalise findings from this study to other populations should be made with caution
				An intrusive measure database	Females were 69% less likely to be restrained than males	Most participants were unique in terms of their mental health needs which limits the generalisation of results	
					50% of males received at least one physical restraint	Staff characteristics and the decision to use intrusive measures could not be assessed from the available data	
Duke et al. (2014), Australia	This project aimed to document the nature and frequency of restrictive interventions used within a child and adolescent psychiatric inpatient unit	Retrospective quality assurance evaluation	Children and adolescent inpatients (mean age 13.8 years; SD 2.9: range 5.8–18) (*n* = 36)	Clinical charts	Interventions were more likely to be used in the first week of admission, in response to physical aggression and in response to aggression occurring in private space	The size of the subsample for outcome analysis was small	The focus was on incident and patient factors, whereas staff factors and institutional characteristics may also be important determinants
				Health of the nation outcome scales for children	Interventions were associated with younger age, male gender, history of aggression, developmental issues, externalising disorder, increased number of diagnoses, and use of psychotropic medication at presentation	The study was conducted in a single unit, limiting generalisability	Interventions were grouped together for analysis, whereas each type may be associated with different predictors
					Factors that remained independently associated with increased likelihood of experiencing an intervention were developmental disorders and younger age	Global symptom ratings are unlikely to capture negative sequelae associated with the use of restrictive interventions	
Furre et al. (2014), Norway	To compare social, mental health, and treatment characteristics of restrained and non-restrained adolescents in acute psychiatric inpatient units	Retrospective case–control study	Adolescent inpatients (13–17 years of age) (*n* = 288)	Characteristics of patient	Restrained group had larger proportions of psychotic, eating, and externalising disorders	Reliability of the clinical data (e.g., diagnoses and CGAS scores) could not be checked due to the retrospective design	The authors provide a solid rationale for the study
				Children’s global assessment scale	Restrained patients had lower psychosocial functioning	Reliability of data extraction was not clarified	The authors also highlight the importance of concomitant pharmacological restraint, recognising the potential impact on this especially vulnerable population
				International statistical classification of diseases (ICD-10) diagnoses	Restrained patients had more and longer admissions and were more often involuntarily referred	No information about missing restraint episodes	
				Restraint vs non-restraint control group			
Muir-Cochrane et al. (2014), Australia	To present an audit report of the use of restrictive measures (seclusion and/or physical restraint) in a child and adolescent mental health unit	Audit report from a retrospective study	Children and adolescent inpatients (3–18 years of age) (*n* = 41)	Non-identifiable incident forms filled out by clinicians at the time of the events	There were more events involving females (59.66%, *n* = 71) than males (40.34%, *n* = 48)	The data were retrospective and are therefore prone to underreporting or missing information	The authors were not able to explore the overall effectiveness of interventions, as no information is available concerning successful interventions that did not include restraint
					Over 50% (*n* =23) of patients had one event only (mdn = 1, mean = 2.90, SD = 3.43), with 21.95% (*n* = 9) having two to three events and 21.95% (*n* = 9) having 4–15 events	Categories used in reporting reasons for containment were general	
					51 events had 1 reason reported (43.22%), 28 had 2 (23.73%), and 39 had 3 (33.05%)		
Furre et al. (2016), Norway	To describe the frequency and duration of restraint use, and reasons for restraining inpatient adolescents	Retrospective analysis	Adolescent inpatients (13–17 years of age) (*n* = 267)	Characteristics of patient	78.7% of restraints were physical	Reliability of the clinical data (e.g., diagnoses and CGAS scores) not checked due to the retrospective design	There is recognition of an absence of a common definition of restraint in practice and how this can make comparisons challenging; however, this study was conducted in a country which is more proactive in conducting research in this field
				CGAS	Restraint reasons: Harming others-53.2%, self-harm-21.7%, damaging property-16.5%, attempted absconsion-13.9%, and acting out-11.2%	Reliability of data extraction was not clarified	
				International statistical classification of diseases (ICD-10) diagnoses		No information about missing restraint episodes	
Furre et al. (2017), Norway	To investigate whether the number of restraint episodes per patient was related to any of several characteristics of the adolescents	Retrospective analysis	Adolescent inpatients (13–17 years of age) (*n* = 267)	Characteristics of patient	97 (36%) adolescents were only restrained once, 88 (33%) 2–4 times, 35 (13%) 5–9 times, and 47 (18%) 10 times or more. This latter group accounted for 1762 (77%) of all restraint episodes	Reliability of the clinical data (e.g., diagnoses and CGAS scores) not checked due to the retrospective design	The relevance of the findings could be discussed in more detail in relation to the aim of this study
				CGAS		Reliability of data extraction was not clarified	There is no discussion of transferability of findings to other populations
				International statistical classification of diseases (ICD-10) diagnoses		No information about missing restraint episodes	

*Note:* CGAS: children's global assessment scale.

### Quality appraisal

Publications were assessed for methodological and reporting biases using the CASP quality assessment tool, assessing for validity, findings, and clinical relevance (Critical Appraisal Skills Programme, 2018). Each publication was reviewed independently by two members of the team. Quality ratings were collated (Table 3), and discrepancies were resolved through academic discussion until consensus was achieved. One publication (Faay et al., 2017) was excluded as the authors’ justification for the study design and data collection was unclear. There was also limited discussion of the study findings, including their reliability and validity and their potential to be transferable to other populations.

**Table 3.** Critical appraisal skills programme quality of evidence.

	Critical appraisal skills programme section										
Author	1 Clear aim	2 Appropriate methodology	3 Appropriate design	4 Appropriate sampling and recruitment	5 Appropriate data collection	6 Consideration of research/participant relationship	7 Consideration of ethical issues	8 Rigorous analysis	9 Findings clearly stated	10 Is the research valuable?	Include?
Sourander et al. (2002)	Y	Y	P	Y	Y	N/A	N	Y	Y	Y	Y
Donovan et al. (2003)	Y	Y	P	Y	Y	N/A	Y	Y	Y	P	Y
Delaney and Fogg (2005)	Y	Y	P	P	Y	N/A	P	Y	P	Y	Y
Leidy et al. (2006)	P	P	Y	Y	Y	N/A	Y	Y	Y	Y	Y
Crocker et al. (2010)	P	Y	Y	P	P	N/A	P	Y	Y	Y	Y
dosReis et al. (2010)	Y	Y	Y	P	Y	N/A	Y	Y	Y	P	Y
Stewart et al. (2010)	Y	Y	P	Y	P	N/A	N	Y	Y	P	Y
Azeem et al. (2011)	Y	Y	Y	P	Y	N/A	P	Y	Y	Y	Y
Tompsett et al. (2011)	Y	Y	Y	Y	Y	Y	Y	Y	Y	Y	Y
Pogge et al. (2013)	Y	Y	Y	Y	Y	N/A	N	Y	Y	P	Y
Stewart et al. (2013)	Y	Y	Y	Y	P	P	Y	Y	Y	Y	Y
Duke et al. (2014)	Y	Y	Y	Y	Y	N/A	N	Y	Y	Y	Y
Furre et al. (2014)	Y	Y	Y	Y	Y	N/A	N	Y	Y	Y	Y
Muir-Cochrane et al. (2014)	Y	Y	Y	Y	Y	N/A	N	Y	Y	Y	Y
Furre et al. (2016)	Y	Y	Y	Y	Y	N/A	N	Y	Y	Y	Y
Faay et al. (2017)	Y	Y	P	P	P	N/A	P	Y	P	P	N
Furre et al. (2017)	Y	Y	Y	Y	Y	N/A	N	Y	Y	Y	Y

Y = yes (fully met criterion), P = partially met criterion, N = no (criterion not met), N/A = not applicable.

### Data synthesis

Narrative synthesis, informed by Popay et al. (2006), facilitated the identification of patterns across the data. Studies were tabulated to allow for preliminary comparison. Relationships within and between the reviewed studies were explored to identify similarities, differences, and potential effects of background variables. Subgroups were derived that best illustrate the emergent themes from within the study findings. Accordingly, these are presented and discussed in respect of the objectives of this review.

## Results

### Study characteristics

Sixteen studies are included in this review representing research conducted across America (*n* = 6), Australia (*n* = 3), Norway (*n* = 3), Canada (*n* = 2), Belgium (*n* = 1), and Finland (*n* = 1). Settings comprise psychiatric inpatient environments (acute and secure units and residential services). Services are representative of both private and state-funded facilities, serving urban and rural populations. Studies were conducted across child and adolescent settings (*n* = 10), adolescent-only environments (*n* = 5; one of which is female only), with one study focussing exclusively on a child-oriented service. Most designs (*n* = 13) are retrospective. All included studies were quantitative in nature, reflecting the paucity of empirical qualitative research in the current literature reporting on the physical restraint of children and adolescents in mental health care. There is evidence of total population sampling (*n* = 5). Two publications provide weak descriptions of sampling and recruitment. Most (*n* = 15) do not acknowledge the relationship between researcher and participant.

Key factors from this review are summarised in Table 4. The reviewed literature indicates that there are some common characteristics associated with children and adolescents who experience restraint within mental health services. The reasons for implementation of restraint include both intrinsic and extrinsic variables and are often derived through complex, dynamic, and reactive decision-making by front-line care providers. Whilst some physical consequences of restraint are reported, psychological consequences are rarely explored. Findings are presented in accordance with the three objectives of this review (which children and adolescents are being physically restrained, the reasons why children and adolescents are reported as being physically restrained, and the reported consequences for children and adolescents of physical restraint use).

**Table 4.** Summary of key factors.

	Key factor
Which children and adolescents are being physically restrained	Age: younger children
	Gender: males (however, females have a greater risk of multiple restraint)
	Diagnosis: children and adolescents with a diagnosed developmental disorder, psychotic disorder, and externalising or internalising disorder (multiple comorbid diagnoses further increase likelihood)
	History: children and adolescents with multiple previous inpatient admissions, history of trauma, self-harm, and aggression
Why children and adolescents are being physically restrained	Risky behaviours: agitation, aggression, threats and staff-directed assault, self-harm, opposition, disinhibition, and absconsion
	Admission status: emergency and voluntary admission status more likely to experience restraint
	Timing: incidents more prevalent at the start of the week, in afternoons or evenings and during longer admission periods; however, incidents generally decrease across an admission period, sometimes after a spike following an initial ‘honeymoon’ period; incidents can ‘cluster’, whereby one physical restraint can spark others
	Staff influences: lack of familiarity with procedures and cultural backgrounds can lead to miscommunication; implementation thresholds can increase over time to only the most dangerous behaviours
Consequences for children and adolescents of physical restraint	Physical injury: there is a potential relationship between physical restraints >15 min and increased risk of physical injury
	Psychological harm: studies associate physical restraint with a lack of therapeutic effect, with implementation potentially worsening behaviours; little is known about adverse psychological effects (such as distress) associated with physical restraint of children and adolescents

### Which children and adolescents are being physically restrained in inpatient mental health services?

Five studies cite that between 27% and 44% of their demographic experienced some type of physical restraint during treatment: 27% (Tompsett et al., 2011), 36.3% (Furre et al., 2016), 38% (Leidy et al., 2006), 40% (Sourander et al., 2002), and 44% (Muir-Cochrane et al., 2014). However, most studies recognise that some incidents of physical restraint use remain underreported (Crocker et al., 2010; Donovan et al., 2003; dosReis et al., 2010; Furre et al., 2014, 2016, 2017; Leidy et al., 2006; Muir-Cochrane et al., 2014; Pogge et al., 2013; Tompsett et al., 2011). dosReis et al. (2010) report that 60% of physically restrained children (*n* = 156) experienced, on average, 14 physical restraints each, potentially identifying a group of children who may be at relatively high risk of physical restraint– and/or psychological restraint–related harm.

#### Association between a child or adolescent’s age and being physically restrained

Younger age (<13 years) is associated with an increased likelihood of physical restraint experience in 10 studies (Azeem et al., 2011; dosReis et al., 2010; Duke et al., 2014; Furre et al., 2014; Leidy et al., 2006; Muir-Cochrane et al., 2014; Pogge et al., 2013; Sourander et al., 2002; Stewart et al., 2010, 2013). Younger children are reported to be more overtly aggressive (Duke et al., 2014; Pogge et al., 2013; Sourander et al., 2002), with aggression being strongly associated with physical restraint. Four studies cite no association between age and physical restraint (Crocker et al., 2010; Delaney and Fogg, 2005; Donovan et al., 2003; Tompsett et al., 2010), although methodological weaknesses are identified in each study (see Table 1). The earliest study (Donovan et al., 2003) excluded interventions lasting less than 15 min, a time period which has subsequently been identified as most common for physical restraint of younger children in this environment (Pogge et al., 2013).

#### Association between a child or adolescent’s gender and being physically restrained

Five studies report that males (regardless of age) are physically restrained significantly more often than females (Delaney and Fogg, 2005; Duke et al., 2014; Stewart et al., 2010, 2013 Sourander et al., 2002), with reported figures of 70% of male children and adolescents being restrained (Duke et al., 2014), whereas three studies report females as having a greater risk of multiple physical restraints than males (dosReis et al., 2010; Furre et al., 2017; Muir-Cochrane et al., 2014). However, the variation in demographics (e.g., age of children and proportion of males to females) means that interpretation is challenging. Crocker et al. (2010) found no identifiable association between gender and physical restraint in their relatively small (*n* = 41) majority male population (78%; *n* = 32).

#### Association between diagnosis/psychosocial functioning and being physically restrained

Restraint incidence is positively associated with children and adolescents with developmental disorders (Azeem et al., 2011; Duke et al., 2014; Sourander et al., 2002), psychotic disorder (Delaney and Fogg, 2005; Furre et al., 2014; Sourander et al., 2002), externalising disorders (conduct/oppositional/disruptive disorders) (Azeem et al., 2011; Crocker et al., 2010; Furre et al., 2014; Leidy et al., 2006; Sourander et al., 2002), and internalising disorders (mood, and depression and anxiety) (Azeem et al., 2011; Delaney and Fogg, 2005; dosReis et al., 2010; Leidy et al., 2006). Crocker et al. (2010), Delaney and Fogg (2005), and Duke et al. (2014) report that having multiple (comorbid) diagnoses further increases the likelihood of the use of physical restraint (comorbidity indicating conditions which coexist in the context of the index condition (Yancik et al., 2007)). Three studies present findings which suggest that it is psychosocial functioning, rather than diagnostic status, which reliably predicts physical restraint experiences (Furre et al., 2014, 2016; Stewart et al., 2013).

#### Association between a child or adolescent’s history and being physically restrained

Physical restraint incidence is positively associated with multiple previous inpatient admissions (Delaney and Fogg, 2005; Furre et al., 2014, 2016), past trauma (Azeem et al., 2011; Delaney and Fogg, 2005; dosReis et al., 2010; Duke et al., 2014; Furre et al., 2014; Tompsett et al., 2011), and history of aggression (Crocker et al., 2010; Duke et al., 2014; Tompsett et al., 2011). Four studies associate a history of self-harm with increased likelihood of physical restraint (Delaney and Fogg, 2005; Furre et al., 2016; Sourander et al., 2002; Stewart et al., 2010).

## Reasons why children and adolescents are reported as being physically restrained in inpatient mental health services

### Intrinsic factors associated with children and adolescents being physically restrained

Crocker et al. (2010) identify how an increase in a child or adolescent’s risky behaviour (presentations associated with aggression and disruptive behaviour disorder) is linked to them being more likely to be physically restrained. Delaney and Fogg (2005) report the most prevalent risky behaviours as agitation, threats, and staff-directed assault (most of which were extremely violent). A child or adolescent’s aggressive acts (defined as harmful behaviour which may include deliberate intent to harm or injure another person (Bandura, 1973, cited in Suris et al., (2004)) most frequently trigger the use of physical restraint (Crocker et al., 2010; Delaney and Fogg, 2005; dosReis et al., 2010; Duke et al., 2014; Furre et al., 2016; Muire-Cochrane et al., 2014; Pogge et al., 2013; Sourander et al., 2002; Stewart et al., 2010, 2013; Tompsett et al., 2011), with child-to-staff aggression being identified as a common precursor (Sourander et al., 2002; Tompsett et al., 2011). Studies also associate the use of physical restraint with ‘lower level’ behaviours (opposition, disinhibition, and absconsion) (Duke et al., 2014; Furre et al., 2016; Muir-Cochrane et al., 2014) as well as destruction of property (Furre et al., 2016; Muir-Cochrane et al., 2014). Self-harming behaviours significantly increase the likelihood of a child or adolescent being physically restrained (Furre et al., 2016; Muir-Cochrane et al., 2014; Stewart et al., 2010), whereas suicidal acts decrease this likelihood in favour of alternative management approaches (Sourander et al., 2002).

### Extrinsic factors associated with children and adolescents being physically restrained

Leidy et al. (2006) found that children and adolescents whose admissions were mandated were twice as likely to experience multiple physical restraints. However, this is contradicted by three studies which report that mandatorily admitted children and adolescents experienced fewer physical restraints than their voluntarily admitted peers (Azeem et al., 2011; Donovan et al., 2003; Furre et al., 2016). Donovan et al. (2003) report that children and adolescents admitted on an emergency basis (52%; *n* = 111) were 4.6 times more likely to experience physical restraint than planned admission peers. Similarly, Crocker et al. (2010) report that 82% of children and adolescents (*n* = 9) admitted as an emergency experienced physical restraint. No article which discussed the status of emergency admission included a clear definition of what constituted an emergency. Although the Department of Health (2013) defines emergency admissions as those which are unpredictable and unplanned, it is not possible to ascertain if this definition aligns with the terminology used in the individual studies within this review.

The literature indicates that the day of the week and the time of the day may influence the prevalence of physical restraint within inpatient mental health settings. Crocker et al. (2010) and Leidy et al. (2006) indicate that physical restraints are more prevalent at the beginning of the week, before steadily decreasing towards Friday. Crocker et al. (2010) identify that fewer incidents occur Friday–Sunday (when most children and adolescents were on home leave), whereas Leidy et al. (2006) report an increase again over the weekend, reinforcing how trends bear relevance only in respect of individual service provision. Four studies (Delaney and Fogg, 2005; dosReis et al., 2010; Furre et al., 2016; Leidy et al., 2006) found that physical restraint is more likely to occur in the afternoon or evening. Two studies found an overall decrease in physical restraints during an admission period (Furre et al., 2016; Leidy et al., 2006), whereas dosReis et al. (2010) reported the opposite, perhaps identifying the existence of an initial ‘honeymoon’ period (where more communicative and/or behavioural effort is expended by children, adolescents, and staff earlier in an admission).

Eight studies claim that longer admission periods are linked to an increase in the likelihood of physical restraint being used (Delaney and Fogg, 2005; dosReis et al., 2010; Furre et al., 2016; Leidy et al., 2006; Pogge et al., 2013; Sourander et al., 2002; Stewart et al., 2013; Tompsett et al., 2011). Whilst the length of stay is an important factor, Leidy et al. (2006) report that its association with physical restraint is not straightforward. Tompsett et al. (2011) offer a practical explanation, suggesting that children and adolescents simply have more time in which to aggress as the length of stay increases. Leidy et al. (2006) identify potential ‘clustering’, 75% of physical restraints (*n* = 794) occurred on less than 20% of the days, with 25% (*n* = 265) occurring on just 3% of the days, potentially highlighting a ‘chain reaction’, whereby one physical restraint has the potential to ‘spark’ others.

Four studies report how multiple interventions were attempted prior to physical restraint (Delaney and Fogg, 2005; Muir-Cochrane et al., 2014; Pogge et al., 2013; Sourander et al., 2002). Delaney and Fogg (2005) report that ‘as-needed’ medication (typically antipsychotic and/or sedative) was used before 38% (*n* = 45) of physical restraint incidents, although the authors report that no further information was available to explore the reasons why physical restraint was subsequently implemented. Five studies show how the use of physical restraint is partly determined by the familiarity of staff with policies (dosReis et al., 2010; Donovan et al., 2003; Furre et al., 2014, 2016, 2017). dosReis et al. (2010) and Donovan et al. (2003) describe how, as time progresses, the threshold for staff using physical restraint increases, with intervention implementation becoming selectively focused on the most dangerous behaviours. Staff members’ lack of familiarity with non-native cultural backgrounds may also lead to misperceptions that some children and adolescents are dangerous, leading to miscommunication and mistrust from both perspectives (dosReis et al., 2010; Furre et al., 2014). Children and adolescents from minority communities have a higher proportion of unmet mental health needs and have often developed more serious symptoms at the point of admission, placing them at a higher risk of physical restraint (Donovan et al., 2003).

### Reported consequences for children and adolescents of physical restraint use in inpatient mental health services

#### Physical consequences of children and adolescents being physically restrained

dosReis et al. (2010) draw attention to the potential for children and adolescents to be exposed to physical (and psychological) harm through increased restraint incidences. Two studies discuss a relationship between longer duration of physical restraint and increased risk of physical harm (Duke et al., 2014; Furre et al., 2016), with Furre et al. (2016) highlighting how restraint duration should be as short as possible to reduce any potentially harmful effects. Donovan et al. (2003) note a reduction of physical restraint frequency (by 26%) and duration (by 38%) over the two-year period of their naturalistic retrospective study. However, there was a proportional increase in the number of children and adolescent’s restraint-related physical injuries (from 4% to 8%) over the same time, suggesting that reduced physical injury risk may not be directly proportional to reduced physical restraint frequency or duration. It is difficult to interpret findings further as physical restraints <15 min were excluded from the study.

#### Psychological consequences of children and adolescents being physically restrained

None of the reviewed studies reported child-reported psychological consequences of physical restraint. Pogge et al. (2013) suggest that some interventions could provide therapeutic benefits, although this is not drawn from empirical evidence. Two studies associate physical restraint with a lack of therapeutic effect (Duke et al., 2014) and a potential to worsen behaviours (Crocker et al., 2010; Duke et al., 2014). Health of the Nation Outcome Scale scores (measuring general health and social functioning) were available for 35% of admitted children (*n* = 47) in the study by Duke et al. (2014), with scores suggesting global improvements across children and adolescent’s admission periods, with no differences between those who did and did not experience physical restraint. However, the authors do not specify if scores were self-rated (by the children or adolescents), parent-rated, or clinician-rated. Further, the authors note that the retrospective dataset did not allow investigation of more immediate adverse effects associated with physical restraint (such as distress). Therefore, this finding should be viewed with some caution.

## Discussion

This is the first systematic review which synthesises research reporting which children and adolescents are being physically restrained in mental health inpatient services, the reasons why, and any potential associated consequences. This review addresses recommendations from an earlier review (DeHert et al., 2011), which called for a focus on indications and predictors, clinical outcomes, and effectiveness of physical restraint of children. Previous systematic reviews have been conducted examining restraint use with adults in mental health care (Dahm et al., 2017; Wilson et al., 2015) and general health care (Mohler et al., 2016) as well as with children in general paediatric care (Bray et al., 2014; Kirwan and Coyne, 2016). There has also been a tendency to approach this subject through examination of the perspectives of nursing staff (Demir, 2007; Kirwan and Coyne, 2016).

The implementation of physical restraint results from complex, dynamic, and reactive decision-making by front-line care providers and can be based upon a combination of patient (intrinsic) and environmental (extrinsic) factors. Some of the more frequently reported characteristics include a child or adolescent’s age, gender, diagnosis, and history, with males restrained significantly more often than females (Delaney and Fogg, 2005; Duke et al., 2014; Stewart et al., 2010, 2013; Sourander et al., 2002) although females can have a greater risk of multiple restraints (dosReis et al., 2010; Furre et al., 2017; Muir-Cochrane et al., 2014). Having a formal diagnosis regularly emerges as a predictor of physical restraint (Azeem et al., 2011; Crocker et al., 2010; Delaney and Fogg, 2005; dosReis et al., 2010; Duke et al., 2014; Furre et al., 2014, 2016; Leidy et al., 2006; Sourander et al., 2002; Stewart et al., 2013). Organisational factors, a child or adolescent’s admission status, longer length of stay, miscommunication, and mistrust of staff (and children) can also contribute to the reasons for physical restraint implementation.

Physical restraint is generally more likely to be used earlier in an admission (dosReis et al., 2010; Furre et al., 2016; Leidy et al., 2006), in the afternoons or evening (Delaney and Fogg, 2005; dosReis et al., 2010; Furre et al., 2016; Leidy et al., 2006). There is also some potential ‘clustering’ of incidents (Leidy et al., 2006). Individually, these characteristics can have predictive merits. In combination, some stronger predictive traits potentially emerge. Several studies reported that multiple interventions were attempted prior to the implementation of restraint, although information concerning positive behavioural interventions (negating restraint use altogether) was rarely reported in the identified literature included within this review. One study excluded interventions lasting less than 15 min (Donovan et al., 2003), although no clear reason was reported for only including interventions where physical restraint lasted longer than 15 min. Contemporary evidence (Mental Health Commission, 2013) shows that 90% of physical restraints (both with children and adult inpatients) last 15 min or less, meaning that if Donovan’s cut-off point was used in current research, most episodes of restraint would be missed. Most studies discuss the physical consequences associated with physical restraint, including physical injury. A focus on physical harm can result in psychological harm (rarely discussed in depth) being overlooked (Bray et al., 2014). There is anecdotal mention of potential therapeutic benefits of physical restraint (Pogge et al., 2013), although the evidence supporting this claim is weak.

DeHert et al. (2011) highlight the importance of the development of restraint guidelines which take into account the input of children. Whilst restraint reduction remains high on the agenda (Department of Health, 2014, 2017), guidelines exist (Ridely and Leitch, 2019), and evaluation of initiatives is important (Caldwell et al., 2014; Valenkamp et al., 2014; Wilson et al., 2015), but the evidence shows that restraint reduction training alone does not necessarily negate the risk of physical injury (Challenging Behaviour Foundation, 2019; Ridely and Leitch, 2019). Restraint implementation with children and adolescents can be enhanced through a richer understanding of the impact of the intervention (Demir, 2007), with credibility existing in the words of those who experience mental health services first-hand (Caldwell et al., 2014). This review has identified that there are no published UK studies focussing on children’s first-hand reported experiences of physical restraint within inpatient child and adolescent mental health services. Services must be able to take into account the individual needs voiced by the children under their care (Caldwell et al., 2014). Therefore, more research is needed to better understand the variables that can lead to restraint (Kirwan and Coyne, 2016) and to listen to what children and adolescents say about their experiences of physical restraint in mental health care.

### Limitations

This review is subject to limitations within the available evidence base. Services are representative of both clinical and residential settings. Although the context and culture of service provision were examined, this was not consistently or effectively reported. However, of those articles (*n* = 11) that did report context, it was clear that some provision was delivered more intensively (dosReis et al., 2003; Leidy et al., 2006; Stewart et al., 2010, 2013) than others (Crocker et al., 2010; Donovan et al., 2003; Furre et al., 2014, 2016, 2017; Sourander et al., 2002; Tompsett et al., 2011). Where resources were available, residential providers appeared positioned to provide more personalised, intensive treatment (Stewart et al., 2010). Some data from clinical providers were reported as more nationally representative (Furre et al., 2017; Sourander et al., 2002), although there is recognition that within both contexts, there is room for improvement on how clinical information is recorded (Furre et al., 2014, 2016, 2017; Leidy et al., 2006), which has the potential to impact review findings. Additionally, there remains a reliance on proxy reporting of physical restraint outcomes for children and adolescents admitted to mental health services. The results of this review rely on a small number of empirical studies, most of which relied on retrospective data which can be prone to missing or unreported data. The study populations differed in terms of age, setting characteristics, and duration of admission periods making some comparisons challenging.

### Recommendations

There is a need to explore children and adolescents’ perceptions about physical restraint and the impact of physical restraint frequency on health outcomes. Future research should continue to assess the use of physical restraint with children and adolescents. Studies should specifically focus upon understanding the effect that physical restraint has upon children and adolescents in mental health care. Studies should also focus on determining, through collaboration with children, the most appropriate use of physical restraint, including the most appropriate approaches for using the intervention when alternative strategies may be more beneficial to the well-being of children, adolescents, and staff.

## Conclusion

This review highlighted some common characteristics associated with children and adolescents who have experienced physical restraint in inpatient mental health care, as well as common reasons for implementation. There remains a reliance on retrospective data, single-centre studies, and proxy reporting of outcomes. This review has identified the absence of first-hand reported accounts of children and adolescents who have experienced physical restraint in inpatient mental healthcare services.
